# Effects of the BDNF Val66Met polymorphism on functional status and disability in young stroke patients

**DOI:** 10.1371/journal.pone.0237033

**Published:** 2020-12-11

**Authors:** Robynne G. Braun, Steven J. Kittner, Kathleen A. Ryan, John W. Cole

**Affiliations:** 1 University of Maryland School of Medicine, Baltimore, MD, United States of America; 2 Veterans Affairs Maryland Health Care System, Baltimore, MD, United States of America; Rutgers University, UNITED STATES

## Abstract

**Background and purpose:**

The preponderance of evidence from recent studies in human subjects supports a negative effect of the BDNF Val66Met polymorphism on motor outcomes and motor recovery. However prior studies have generally reported the effect of the Met allele in older stroke patients, while potential effects in younger stroke patients have remained essentially unexamined. The lack of research in younger patients is significant since aging effects on CNS repair and functional recovery after stroke are known to interact with the effects of genetic polymorphisms. Here we present a study of first-ever ischemic stroke patients aged 15–49 years that examines the effect of Met carrier status on functional disability.

**Methods:**

829 patients with a first ischemic stroke (Average age = 41.4 years, SD = 6.9) were recruited from the Baltimore-Washington region. Genotyping was performed at the Johns Hopkins University Center for Inherited Disease Research (CIDR). Data cleaning and harmonization were done at the GEI-funded GENEVA Coordinating Center at the University of Washington. Our sample contained 165 Met carriers and 664 non-Met carriers. Modified Rankin scores as recorded at discharge were obtained from the hospital records by study personnel blinded to genotype, and binarized into “Good” versus “Poor” outcomes (mRS 0–2 vs. 3+), with mRS scores 3+ reflecting a degree of disability that causes loss of independence.

**Results:**

Our analysis showed that the Met allele conveyed a proportionally greater risk for poor outcomes and disability-related loss of independence with mRS scores 3+ (adjusted OR 1.73, 95% CI 1.13–2.64, *p* = 0.01).

**Conclusions:**

The *BDNF* Val66Met polymorphism was negatively associated with functional outcomes at discharge in our sample of 829 young stroke patients. This finding stands in contrast to what would be predicted under the tenets of the resource modulation hypothesis (i.e. that younger patients would be spared from the negative effect of the Met allele on recovery since it is posited to arise as a manifestation of age-related decline in physiologic resources).

## Introduction

It is a common observation that the effects of genetic variations become more prominent as the brain’s physiologic resources decline with age. This interaction between aging and genetic polymorphisms has been referred to as the “resource modulation hypothesis”, first articulated by Lindenberger et al in 2008 [1]. Several recent studies [2–4] have exemplified interactions of this sort for a common single nucleotide polymorphism (SNP) referred to as Val66Met, a missense mutation in the pro-domain of *BDNF* that leads to a methionine (Met) substitution for valine (Val) at codon 66. Multiple prior studies have examined the effects of the Val66Met polymorphism in older stroke patients, however, the effect of this polymorphism in younger stroke patients has been less well-studied. To address this knowledge gap, here we examined the effects of the Val66Met polymorphism on post-stroke functional outcomes in early-onset ischemic stroke patients aged 15–49 years.

## Methods

Full details of the experimental protocol are reported elsewhere [5]. In brief, 829 participants with first ischemic stroke aged 15–49 years (average = 41.4, SD = 6.9) were recruited from the Baltimore-Washington region. Genotyping was performed at the Johns Hopkins University Center for Inherited Disease Research (CIDR). Data cleaning and harmonization were done at the GEI-funded GENEVA Coordinating Center at the University of Washington. These data had been collected previously at our clinical site as part of the “GENEVA” initiative (Gene Environment Association Studies) and “GEOS” study (Genetics of Early Onset Stroke). Modified Rankin scores at the time of discharge from the acute hospital were obtained from hospital records by raters blinded to the results of the genetic data. Scores were binarized for analysis into “Good” versus “Poor” outcomes (mRS 0–2 vs. 3+, respectively). We performed a case-only analysis, fitting a logistic regression model that included age, gender, and principle components to the data. This was constructed as an additive model to account for potential gene “dosage” effects (i.e. Val-Met vs. Met-Met). We did not model treatment effects (e.g. for thrombolysis), since the allocation of a given allele is random in the study population and therefore is not influenced by treatment factors. This study was conducted with the consent of all study subjects and was approved by the University of Maryland at Baltimore Institutional Review Board.

## Results

Our sample contained 165 Met carriers and 664 non-Met carriers. **Table 1** details patient characteristics and risk factors. **Table 2** shows the association of Met carrier status with outcome, in the entire population and as stratified by ethnicity and gender. Analysis of the full sample comprising 829 cases revealed a significant association between the Met allele and mRS outcomes, where the Met allele conveyed a proportionally greater risk for poor mRS outcomes (adjusted OR 1.46, 95% CI 1.003–2.137, *p* = 0.05). When length of stay was added to the model (for n = 805 cases with available length-of-stay data) both the magnitude and significance of the association increased (adjusted OR 1.73, 95% CI 1.13–2.64, *p* = 0.01), likely because some of the random, non-genetic influences on outcome were removed. The effect was in the same direction across both ethnicities, and for males and females, but only reached significance in the male group. There was no significant difference in the proportion of stroke subtypes (i.e. lacunar, cardioembolic, atherosclerotic or indeterminate) within each genotype.

**Table 1:** Age, sex, race and risk factor characteristics.

	Cases (n = 829)
**Age**	
Mean age (years)	41.5 ± 6.9
**Sex**	
Female (%)	41.8
Male (%)	58.2
**Self-Reported Race (%)**	
European Ancestry (EA)	52.8
African Ancestry (AA)	42.2
Other	5.1
**Stroke Risk Factors**	
Hypertension (%)	43.1
Diabetes mellitus (%)	16.9
Current smokers (%)	42.1
Angina/MI (%)	5.5
≥ 1 Vascular Risk Factor* (%)	69.2

*Vascular risk factors: hypertension, diabetes mellitus, current smoking, angina/MI.

**Table 2:** Results of case-only analysis using an additive model that accounted for sex, age, and principal components.

	n (Met/Non-Met)	Odds Ratio (mRS 0–2 vs. 3+)	*p*	*CI*
All cases	829	0.68	0.05	0.47–0.997
Ethnicity				
European	448	0.73	0.14	0.48–1.11
African-American	381	0.50	0.11	0.21–1.16
Gender				
Male	482	0.57	0.03	0.34–0.95
Female	347	0.80	0.43	0.49–1.41

## Discussion

BDNF plays a critical role in nervous system development and function, and perturbations in BDNF trafficking can lead to impairments in CNS function. The Val66Met polymorphism affects a region of the BDNF prodomain that interacts with secretory machinery, leading to reduced activity-dependent secretion of BDNF [6] and system-level motor function effects including reduced brain activation in the primary sensorimotor cortex contralateral to the moving limb [7].

Our analysis showed that the *BDNF* Val66Met polymorphism was negatively associated with functional outcomes at discharge in our cadre of young stroke patients. In general, the preponderance of evidence from recent studies in human subjects has supported a *negative effect* of the Met allele on motor outcomes and motor recovery. For example Kim et al. showed poorer motor outcomes in Met carriers on the Fugl-Meyer at 30 and 90 days [8]. Helm et al. [9] and Charalambous et al. [10] found a slower rate of motor adaptation in Met carriers. Shiner et al. reported reduced post-therapy motor improvement in Met carriers with moderate to high motor function [11]. Van der Vliet et al. showed diminished motor learning for Met carriers [12]. Apart from these behavioral studies, negative effects are also reported in neuroimaging and neurostimulation studies. For example, Kim et al. showed decreased brain activation in Met carriers with hemiparesis 12 months post-stroke [7], and Fridriksson et al. showed that Met carriers were less likely to benefit from A-tDCS for aphasia [13]. Some studies, however, have reported *no effect* of the polymorphism on motor outcomes or treatment response. For example, French et al. demonstrated that the *BDNF* genotype did not account for differences in functional mobility at 6 months [14]. Others have even reported the *opposite effect*, including Quin et al. [15] who reported an adaptive role for the Met allele in their rodent model of stroke, and Mirowska-Guzel et al. who showed less favorable stroke rehabilitation outcomes in *non*-Met carriers in the first 90 days post stroke [16].

This inconsistency in the direction of findings across studies could be attributed to many factors, including ethnicity, gene-gene interactions, stroke severity, or—germane to the present study—variations in age. Age effects can furthermore have sex-specific interactions due to changes in hormone expression across the lifespan. Our analysis showed a significant effect of the Met allele in males but not females, and while it is possible that sample size alone accounts for this finding (n = 482 males vs. 347 females), the potential for a protective effect of estrogen in this sample of young females also warrants comment. Sex differences in brain BDNF expression have previously been shown in animal studies to vary with estradiol levels [17]. Furthermore in human studies, plasma BDNF levels are higher during the luteal phase of the menstrual cycle [18]. The effects of estrogen on stroke risk and stroke recovery are complex, with known protective effects of 17β-estradiol on stroke risk for women compared to men [19], but also increased stroke risk with high estrogen levels during pregnancy or with the use of high-estrogen oral contraceptives. Estrogen effects on stroke severity and stroke recovery are less well-studied. The possibility that the BDNF Val66Met polymorphism may have a sexually dimorphic effect on stroke recovery is thus an important consideration in study design.

A limitation to the interpretation of our findings is that baseline NIHSS and mRS data were not available for all patients in our sample, hence the possibility that initial stroke severity was greater for Met carriers, or that pre-hospitalization functional deficits were present, cannot be entirely ruled out. The lack of appropriate baseline and longitudinal measures in large data sets is a key methodological concern for stroke outcomes research in general, with such data frequently missing from clinical stroke registries and administrative databases. For example, despite its well-established clinical significance, NIHSS documentation in the first 5 years of the Get With The Guidelines-Stroke Program (GWTG) was missing in 60.3% of cases [20]. The significance of this problem is now becoming increasingly clear as the field of stroke genetics continues to expand beyond its initial focus on factors affecting stroke *risk* to those factors affecting stroke *recovery*. While stroke *outcomes* can be measured at a discrete time point without consideration of baseline status, *recovery per se* is a relative measure and necessitates reference to a defined baseline. Recognizing the critical significance of this issue for stroke recovery and rehabilitation trials, our research group is actively engaged with the International Stroke Genetics Consortium Global Alliance to generate a consensus statement outlining common data elements and data collection timepoints for appropriate baseline and longitudinal measures in stroke genetics studies (manuscript in review).

In closing, to our knowledge this is the first study to analyze the effects of the *BDNF* Val66Met polymorphism in early-onset stroke patients. The average age across a sample of recent studies was 60.3 years (SD 9.4), while the average age in our sample was 41.9 years (SD 6.8). Our finding that the Met allele carried a proportionally greater risk for poor mRS outcomes among young stroke survivors stands in contrast to what would be predicted under the tenets of the resource modulation hypothesis (i.e. that younger patients would be spared from the negative effect of the Met allele on recovery since it is posited to arise as a manifestation of age-related decline in physiologic resources). (1–4) This novel finding warrants replication in other cohorts of early-onset stroke subjects.

## References

[1] Lindenberger U, Nagel IE, Chicherio C, Li S-C, Heekeren HR, Bäckman L. Age-related decline in brain resources modulates genetic effects on cognitive functioning. Front Neurosci. 2008;2: 234–244. 10.3389/neuro.01.039.2008 19225597 PMC2622748

[2] Tsai S-J. Critical Issues in BDNF Val66Met Genetic Studies of Neuropsychiatric Disorders. Front Mol Neurosci. 2018;11 10.3389/fnmol.2018.00156 29867348 PMC5962780

[3] Kennedy KM, Reese ED, Horn MM, Sizemore AN, Unni AK, Meerbrey ME, et al BDNF val66met polymorphism affects aging of multiple types of memory. Brain Res. 2015;1612: 104–117. 10.1016/j.brainres.2014.09.044 25264352 PMC4377126

[4] Balkaya M, Cho S. Genetics of stroke recovery: BDNF val66met polymorphism in stroke recovery and its interaction with aging. Neurobiol Dis. 2018 10.1016/j.nbd.2018.08.009 30118755 PMC7653673

[5] Cheng Y-C, O’Connell JR, Cole JW, Stine OC, Dueker N, McArdle PF, et al Genome-Wide Association Analysis of Ischemic Stroke in Young Adults. G3 (Bethesda). 2011;1: 505–514. 10.1534/g3.111.001164 22384361 PMC3276159

[6] Egan MF, Kojima M, Callicott JH, Goldberg TE, Kolachana BS, Bertolino A, et al The BDNF val66met polymorphism affects activity-dependent secretion of BDNF and human memory and hippocampal function. Cell. 2003;112: 257–269. 10.1016/s0092-8674(03)00035-7 12553913

[7] Kim DY, Quinlan EB, Gramer R, Cramer SC. BDNF Val66Met Polymorphism Is Related to Motor System Function After Stroke. Phys Ther. 2016;96: 533–539. 10.2522/ptj.20150135 26381810 PMC4817211

[8] Kim E-J, Park C-H, Chang WH, Lee A, Kim ST, Shin Y-I, et al The brain-derived neurotrophic factor Val66Met polymorphism and degeneration of the corticospinal tract after stroke: a diffusion tensor imaging study. Eur J Neurol. 2016;23: 76–84. 10.1111/ene.12791 26228236

[9] Helm EE, Tyrell CM, Pohlig RT, Brady LD, Reisman DS. The presence of a single-nucleotide polymorphism in the BDNF gene affects the rate of locomotor adaptation after stroke. Exp Brain Res. 2016;234: 341–351. 10.1007/s00221-015-4465-8 26487176 PMC4838573

[10] Charalambous CC, Alcantara CC, French MA, Li X, Matt KS, Kim HE, et al A single exercise bout and locomotor learning after stroke: physiological, behavioural, and computational outcomes. J Physiol (Lond). 2018;596: 1999–2016. 10.1113/JP275881 29569729 PMC5978382

[11] Shiner CT, Pierce KD, Thompson-Butel AG, Trinh T, Schofield PR, McNulty PA. BDNF Genotype Interacts with Motor Function to Influence Rehabilitation Responsiveness Poststroke. Front Neurol. 2016;7: 69 10.3389/fneur.2016.00069 27242654 PMC4868962

[12] van der Vliet R, Ribbers GM, Vandermeeren Y, Frens MA, Selles RW. BDNF Val66Met but not transcranial direct current stimulation affects motor learning after stroke. Brain Stimul. 2017;10: 882–892. 10.1016/j.brs.2017.07.004 28751226

[13] Fridriksson J, Elm J, Stark BC, Basilakos A, Rorden C, Sen S, et al BDNF genotype and tDCS interaction in aphasia treatment. Brain Stimul. 2018;11: 1276–1281. 10.1016/j.brs.2018.08.009 30150003 PMC6293970

[14] French MA, Morton SM, Pohlig RT, Reisman DS. The relationship between BDNF Val66Met polymorphism and functional mobility in chronic stroke survivors. Top Stroke Rehabil. 2018;25: 276–280. 10.1080/10749357.2018.1437938 29480080 PMC5901741

[15] Qin L, Jing D, Parauda S, Carmel J, Ratan RR, Lee FS, et al An adaptive role for BDNF Val66Met polymorphism in motor recovery in chronic stroke. J Neurosci. 2014;34: 2493–2502. 10.1523/JNEUROSCI.4140-13.2014 24523540 PMC3921423

[16] Mirowska-Guzel D, Gromadzka G, Mendel T, Janus-Laszuk B, Dzierka J, Sarzynska-Dlugosz I, et al Impact of BDNF -196 G>A and BDNF -270 C>T polymorphisms on stroke rehabilitation outcome: sex and age differences. Top Stroke Rehabil. 2014;21 Suppl 1: S33–41. 10.1310/tsr21S1-S33 24722042

[17] Spencer-Segal JL, Waters EM, Bath KG, Chao MV, McEwen BS, Milner TA. Distribution of Phosphorylated TrkB Receptor in the Mouse Hippocampal Formation Depends on Sex and Estrous Cycle Stage. J Neurosci. 2011;31: 6780–6790. 10.1523/JNEUROSCI.0910-11.2011 21543608 PMC3108038

[18] Begliuomini S, Casarosa E, Pluchino N, Lenzi E, Centofanti M, Freschi L, et al Influence of endogenous and exogenous sex hormones on plasma brain-derived neurotrophic factor. Hum Reprod. 2007;22: 995–1002. 10.1093/humrep/del479 17251358

[19] Koellhoffer EC, McCullough LD. The Effects of Estrogen in Ischemic Stroke. Transl Stroke Res. 2013;4: 390–401. 10.1007/s12975-012-0230-5 24323337 PMC4275797

[20] Smith EE, Shobha N, Dai D, Olson DM, Reeves MJ, Saver JL, et al Risk score for in-hospital ischemic stroke mortality derived and validated within the Get With the Guidelines-Stroke Program. Circulation. 2010;122: 1496–1504. 10.1161/CIRCULATIONAHA.109.932822 20876438

